# Supplementary material for: Androgen deprivation induces double-null prostate cancer via aberrant nuclear export and ribosomal biogenesis through HGF and Wnt activation
Source: Nat Commun. 2024 Feb 9;15:1231. doi: 10.1038/s41467-024-45489-4 (PMC10858246; doi:10.1038/s41467-024-45489-4)
Supplement: Supplementary file 1 — Supplementary Information [file 41467_2024_45489_MOESM1_ESM.pdf]

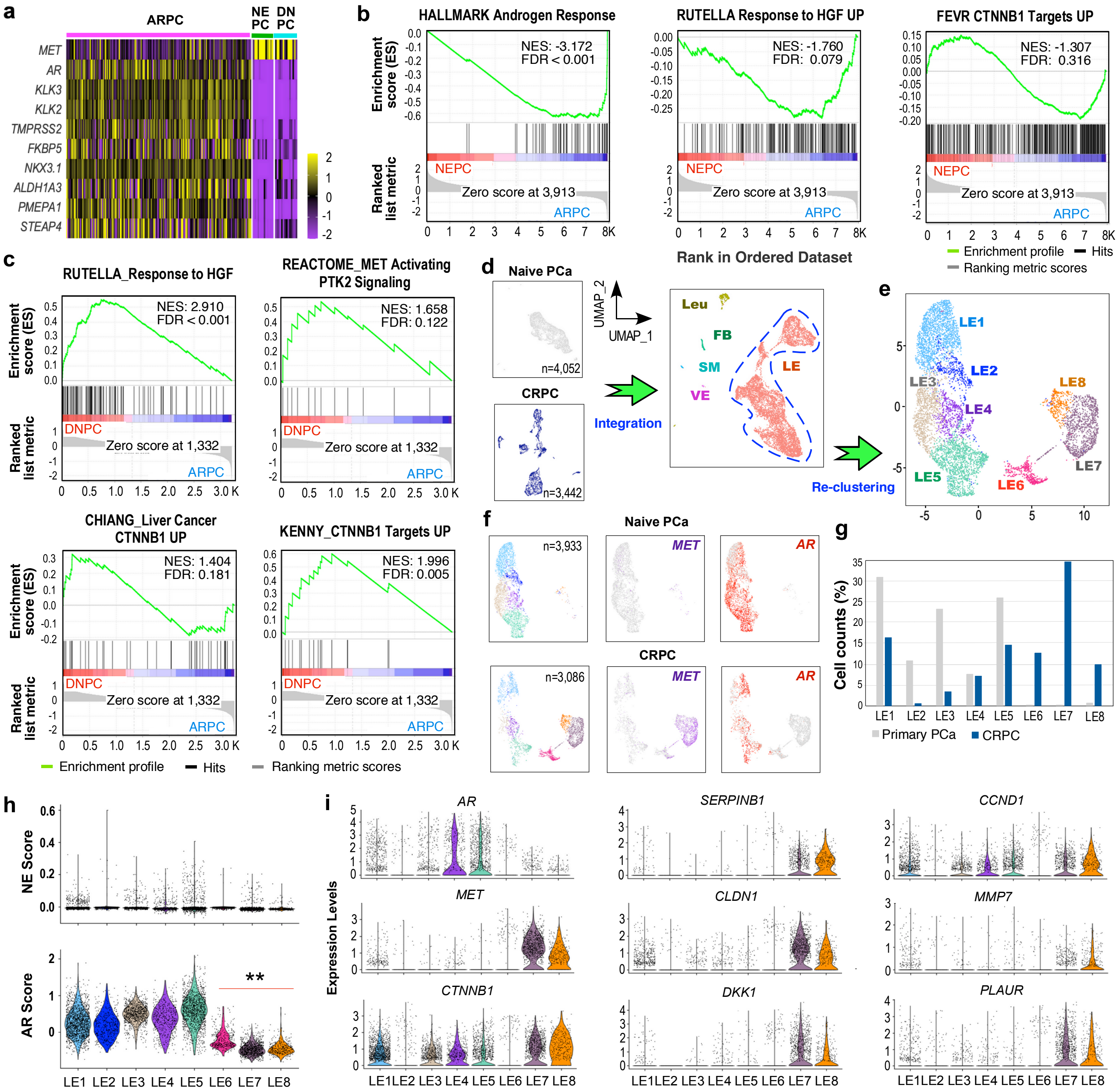

Supplementary Fig. 1 (Related to Fig. 1) Activated MET and Wnt signaling pathways in ADT-treated CRPC patients. a Heatmap showing expression profile of MET, androgen receptor (AR), and AR downstream target genes across human metastatic castration-resistant prostate cancer (CRPC) samples obtained from SU2C/PCF Dream Team datasets using cBioPortal (ARPC, AR-active prostate cancers, n = 177; NEPC, neuroendocrine prostate cancers, n = 13; DNPC, double-negative AR-null neuroendocrine (NE)-null prostate cancers, n = 14). Colors reflect the level of expression. See Methods section. **b** and **c** Gene Set Enrichment Analysis (GSEA) enrichment plots of pre-ranked gene list from differentially expressed genes (DEGs) comparing NEPC (**b**) or DNPC (**c**) to ARPC. NES, normalized enrichment score; FDR, false discovery rate. See also Supplementary Data 1. **d** Uniform Manifold Approximation and Projection (UMAP) visualization of 4,052 and 3,442 individual cells from naive prostate cancer (PCa; top left, gray) and androgen deprivation therapy (ADT)-treated CRPC (bottom left, dark blue) patient samples, respectively, deposited in NCBI's SRA database through PRJNA699369, and integrated cells colored by cell type identities (right). The dotted line delineated the prostatic luminal epithelial cells. LE, luminal epithelial cells; FB, fibroblasts; SM, smooth muscle cells; VE, vascular endothelial cells; Leu, leukocytes. **e** UMAP plot of LE cells sub-clustered, re-clustered, and labeled by cell type. **f** UMAP plots separated by PCa and CRPC LE cells, and expression UMAP plots showing levels of *MET* and *AR*. Purple or red color intensity indicates the scaled expression level in each cell. **g** Bar chart showing the cell counts within individual clusters from LE of PCa and CRPC samples. In **h** and **i,** violin plots visualizing the scores of NE or AR gene signature (**h**) and the expression levels of indicated genes (**i**) in LE clusters (LE1, n = 236; LE2, n = 385; LE3, n = 200; LE4, n = 555; LE5, n = 608; LE6, n = 160; LE7, n = 340; LE8, n = 504) of integrated sample. **P < 0.01; Wilcoxon Rank Sum tests. Black dots correspond to individual LE cells. Source data are provided in the Source Data file.

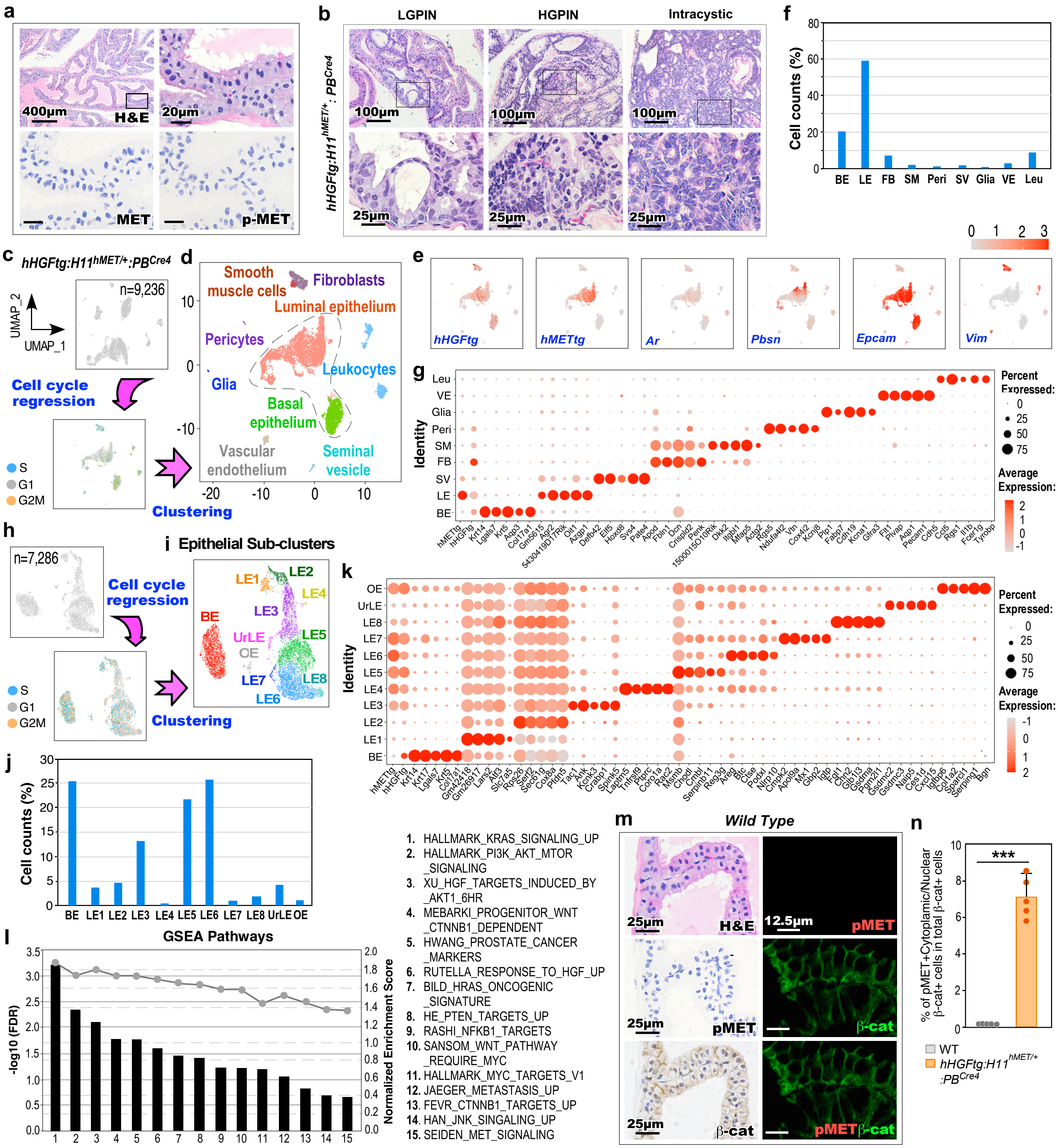

Supplementary Fig. 2 (Related to Fig. 2) HGF/MET signaling-induced aberrant activation of Wnt pathway promotes prostate oncogenesis. a Representative images of H&E and IHC staining using prostates from *wild-type* mice. **b** Representative images of H&E staining for indicated prostate lesions in *hHGFtg:H11*$^{hMET/+}$*:PB*^{Cre4} mice. **c** UMAP plots of total cells from *hHGFtg:H11*$^{hMET/+}$*:PB*^{Cre4} colored by gray before cell cycle regression (top) and cell cycle identity after cell cycle scoring and regression (bottom). **d** UMAP visualization of 9,236 individual cells colored and labeled by cell type. **e** Gene expression UMAP plots for the indicated genes in total cells from *hHGFtg:H11*$^{hMET/+}$*:PB*^{Cre4} mice. Color intensity indicates the scaled expression level in each cell. **f** Bar chart showing the cell counts within individual clusters from total cells. BE, basal epithelial cells; LE, luminal epithelial cells; SV, seminal vesicle; FB, fibroblasts; SM, smooth muscle cells; Peri, pericytes; Glia, glial cells; VE, vascular endothelial cells; Leu, leukocytes. **g** Dot plot of *hMETtg*, *hHGFtg*, as well as five cluster-specific genes for each total cell cluster. Dot size indicates the percentage of cells in a cluster expressing each gene; color intensity corresponds to expression level. **h** UMAP plots of epithelial cells from **d** colored by gray before cell cycle regression (top left), cell cycle identity after cell cycle scoring and regression (bottom), and **i** cell type identity (top right). **j** Bar chart showing the cell counts within individual clusters from epithelial cells. **k** Dot plot of *hMETtg*, *hHGFtg*, as well as five cluster-specific genes for each epithelial cell cluster. Dot size indicates the percentage of cells in a cluster expressing each gene; color intensity corresponds to expression level. **l** GSEA results from pre-ranked DEG list comparing *hMETtg+* versus *hMETtg-* epithelial cells from *hHGFtg:H11*$^{hMET/+}$*:PB*^{Cre4} samples. FDR, false discovery rate. See Supplementary Data 2. **m** Representative images of H&E, IHC, and IF using indicated antibodies on adjacent prostate tissues section from *wild-type* mice. **n** Quantification graph of p-MET- and cytosolic or nuclear β-cat-positive cells in total β-cat-positive cells in prostate tissues from *hHGFtg:H11*$^{hMET/+}$*:PB*^{Cre4} and *wild-type* (WT) mice. Data are mean ± s.d. of five biological replicates. ***P < 0.01; Unpaired two-tailed t-tests. In **a**, **b**, and **m,** Representative images from at least two biological replicates are shown. Scale bars, 400 µm (**a**), 100 µm (**b**), 25 µm (**b**, **m**), 20 µm (**a**), 12.5 µm (**m**). Source data and the exact P values are provided in the Source Data file.

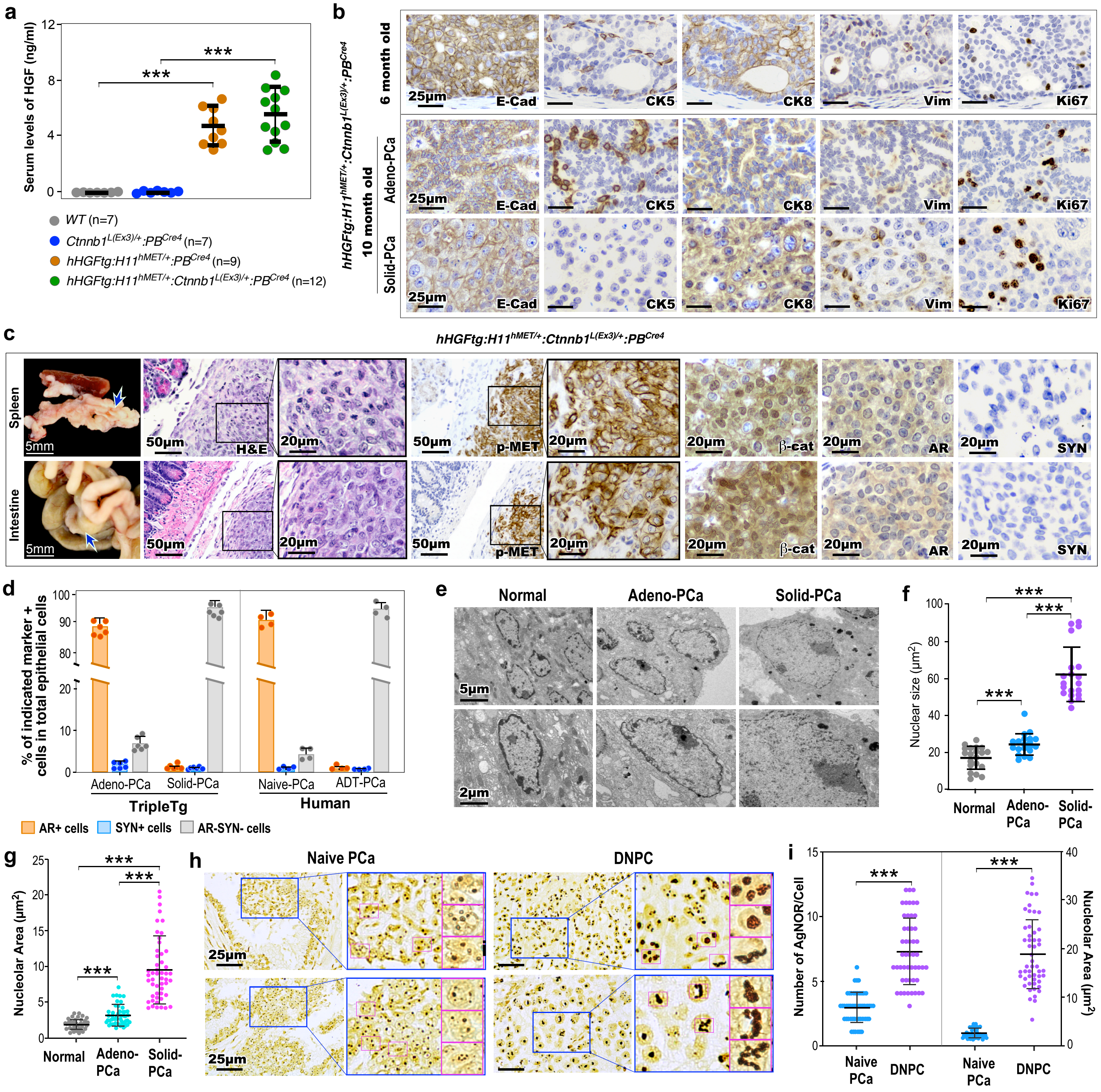

Supplementary Fig. 3 (Related to Fig. 3) Metastatic and aggressive DNPC development induced by HGF/MET and Wnt signaling activation. a Serum HGF concentration from mice of indicated genotype. **b** Representative images of immunohistochemistry (IHC) staining using indicated antibodies on adjacent prostate tissue sections from *hHGFtg:H11*$^{hMET/+}$*:Ctnnb1*$^{L(Ex3)/+}$*:PB*^{Cre4} mice at indicated age. **c** Representative images of hematoxylin & eosin (H&E) and IHC staining using the indicated antibodies on adjacent tissues of pancreas (top) and intestine (bottom) from *hHGFtg:H11*$^{hMET/+}$*:Ctnnb1*$^{L(Ex3)/+}$*:PB*^{Cre4} mice at 10 months of age. **d** Quantification of positive cells for indicated marker per total epithelial cells from *hHGFtg:H11*$^{hMET/+}$*:Ctnnb1*$^{L(Ex3)/+}$*:PB*^{Cre4} mice (n = 6) and naïve primary prostate adenocarcinoma (PCa) and androgen deprivation therapy (ADT)-treated CRPC patients (n = 4). **e** Representative images of transmission electron microscopy in the indicated lesions from *hHGFtg:H11*$^{hMET/+}$*:Ctnnb1*$^{L(Ex3)/+}$*:PB*^{Cre4} prostates. **f** Quantification of nuclear size per cell in the indicated lesions from prostate tissues. 20 cells were analyzed for each locus from at least five images of three biological replicates. **g** Quantification of nucleolar area in the indicated lesions from prostate tissues. **h**, Representative images of AgNOR stained prostate tissue sections from naïve primary prostate adenocarcinoma (PCa) and androgen deprivation therapy (ADT)-treated DNPC patients. **i** Quantification of AgNOR number and nucleolar area in individual cells from the indicated loci. In **a**, **d**, **f**, **g**, and **i**, data are mean ± s.d. of at least three biological replicates. ***$P < 0.001$; Unpaired two-tailed *t*-tests. In **b**, **c**, **e**, and **h**, representative images with consistent results from three biologically independent experiments are shown. In **g** and **i**, nucleolar area or number of AgNORs per cells were measured for 50 cells in each image, and at least five images from three biological replicates were analyzed. Scale bars, 5 mm (**c**), 50 μm (**c**), 25 μm (**b**), 20 μm (**c**), 5 μm (**e**), 2 μm (**e**). Source data and the exact *P* values are provided in the Source Data file.

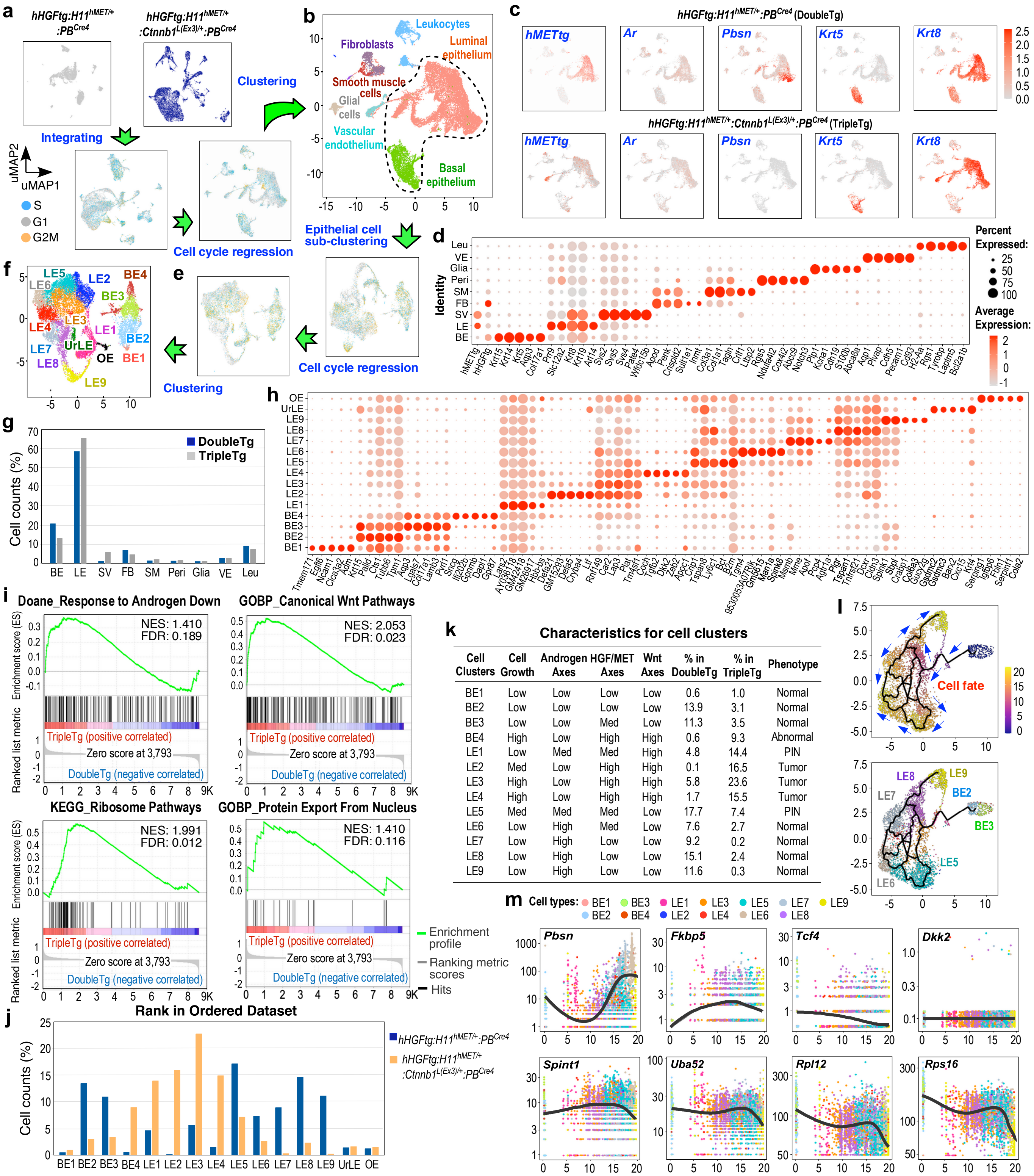

Characteristics for cell clusters

Cell Clusters	Cell Growth	Androgen Axes	HGF/MET Axes	Wnt Axes	% in DoubleTg	% in TripleTg	Phenotype
BE1	Low	Low	Low	Low	0.6	1.0	Normal
BE2	Low	Low	Low	Low	13.9	3.1	Normal
BE3	Low	Low	Med	Low	11.3	3.5	Normal
BE4	High	Low	High	High	0.6	9.3	Abnormal
LE1	Low	Med	Med	High	4.8	14.4	PIN
LE2	Med	Low	High	High	0.1	16.5	Tumor
LE3	High	Low	High	High	5.8	23.6	Tumor
LE4	High	High	High	High	1.7	15.5	Tumor
LE5	Med	Med	Med	Low	17.7	7.4	PIN
LE6	Low	High	Med	Low	7.6	2.7	Normal
LE7	Low	High	Low	Low	9.2	0.2	Normal
LE8	Low	High	High	Low	15.1	2.4	Normal
LE9	Low	High	Low	Low	11.6	0.3	Normal

Supplementary Fig. 4 (Related to Fig. 4) Activated nuclear export signals and ribosomal biogenesis in aggressive prostate cancer cells. **a** Individual UMAP visualization of cells from *hHGFtg:H11$^{hMET/+}$:PB^{Cre4}* prostate (top left, gray, n = 9,236) and from *hHGFtg:H11$^{hMET/+}$:Ctnnb1$^{L(Ex3)/+}$:PB^{Cre4}* prostate (top right, dark blue, n = 9,492), and integrated cells colored by cell cycle identity before (bottom left) and after cell cycle regression (bottom right). **b** UMAP visualization showing cell-type cluster identities based on gene expression profiles. The dotted line delineates the prostatic epithelial cells. **c** UMAP plots separated by indicated genotype (DoubleTg, n = 9,236; TripleTg, n = 9,492) from b and gene expression levels. Color intensity indicates the scaled expression in each cell. **d** Dot plot of *hMETtg*, *hHGFtg*, as well as five cluster-specific genes for each total cell cluster. Dot size indicates the percentage of cells in a cluster expressing each gene; color intensity corresponds to expression level. BE, basal epithelial cells; LE, luminal epithelial cells; SV, seminal vesicle; FB, fibroblasts; SM, smooth muscle cells; Peri, pericytes; Glia, glial cells; VE, vascular endothelial cells; Leu, leukocytes. **e** UMAP plots of epithelial cells sub-clustered, re-clustered, and colored by cell cycle identity before (right) and after cell cycle regression (left). **f** UMAP visualization of epithelial cells, labeled by epithelial cluster. UrLE, urethral luminal epithelial cells; OE, other epithelial cells. **g** Distribution of total cells for each cell type in each of indicated genotypes. **h** Dot plot depicting five specific genes in each subtype from epithelial cells. **i** GSEA enrichment plot depicting indicated pathway significantly up-regulated in *hMETtg*+ cells from *hHGFtg:H11$^{hMET/+}$:Ctnnb1$^{L(Ex3)/+}$:PB^{Cre4}* versus from *hHGFtg:H11$^{hMET/+}$:PB^{Cre4}* mice. See Supplementary Data 3. **j** Distribution of epithelial cells for each cell cluster in each indicated genotypes. **k** Table summarizing the characteristics for each epithelial cell cluster from **f**. **l** Pseudotime trajectory plots displaying a predicted directional path of *hMETtg*+ epithelial cells (n = 4,417) from *hHGFtg:H11$^{hMET/+}$:PB^{Cre4}* mice, visualized on UMAP and colored by pseudotime (top) or cluster identity (bottom). **m** Linear pseudotime expression plots showing dynamics of expression over pseudotime of indicated genes in *hMETtg*+ epithelial cells from *hHGFtg:H11$^{hMET/+}$:PB^{Cre4}* mice. Dots correspond to individual cells colored by cell type identity. Source data are provided in the Source Data file.

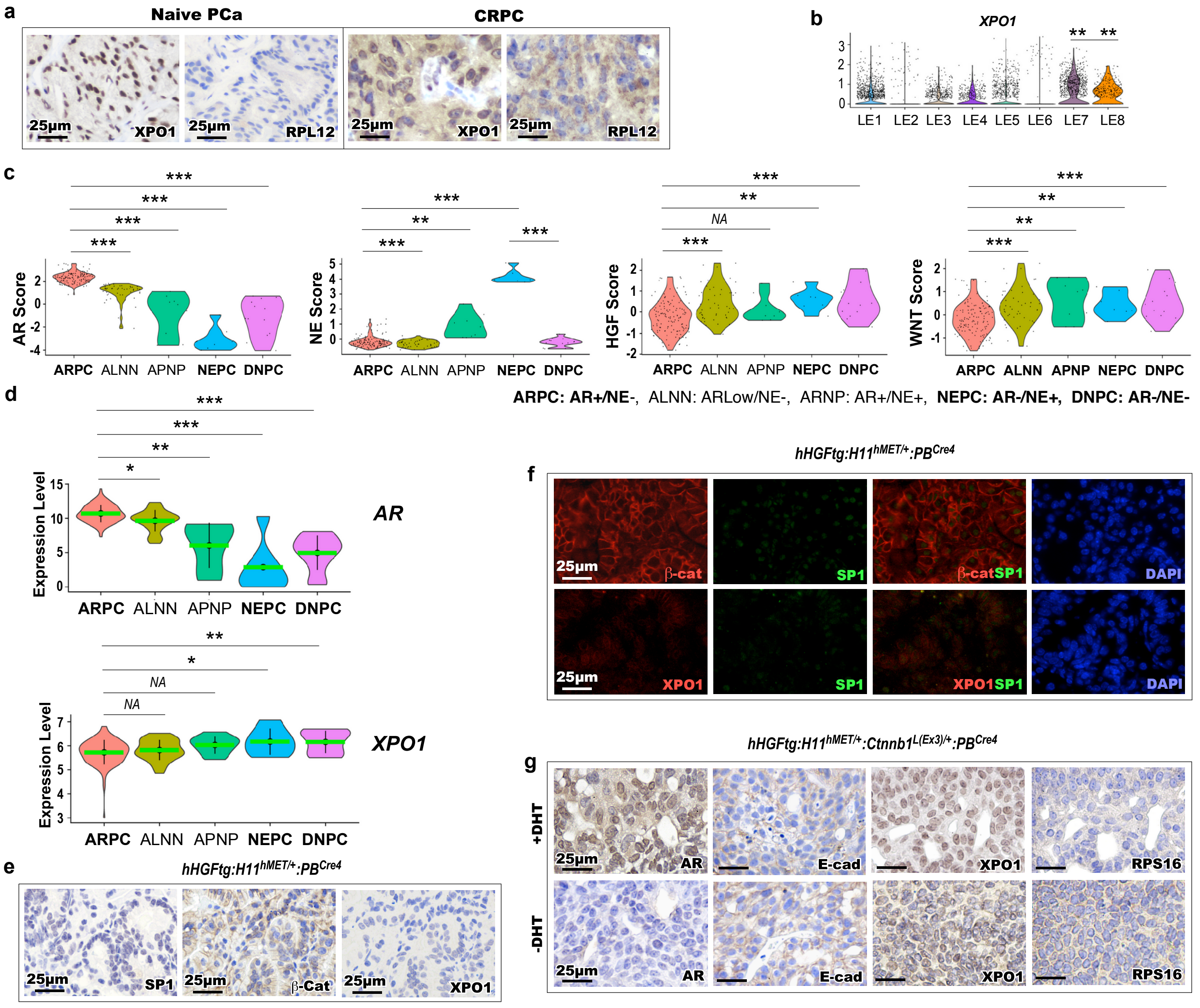

Supplementary Fig. 5 (Related to Fig. 5) β-catenin up-regulates XPO1 expression and activity by increasing SP1 expression in aggressive prostate cancer. **a** Representative images of IHC for XPO1 and RPL12 on tissue sections from naive primary prostate cancer (PCa; left) and ADT-treated castration-resistant prostate cancer (CRPC; right) patients. **b** Violin plots visualizing the expression levels of *XPO1* in human mCRPC luminal cell clusters LE1-LE8 (please also see Supplementary Fig. 1). Black dots correspond to individual luminal cells (LE1, n = 236; LE2, n = 385; LE3, n = 200; LE4, n = 555; LE5, n = 608; LE6, n = 160; LE7, n = 340; LE8, n = 504). **c** and **d** Violin plots visualizing expression of downstream targets for indicated signaling pathway (**c**) and expression levels of *AR* and *XPO1* (**d**) in human mCRPC subtypes (AR+/NE−, ARPC, n = 132; AR+/NE+, ARNP, n = 9; ARL/NE−, ALNN, n = 49; AR−/NE+, NEPC, n = 7; AR−/NE−, DNPC, n = 13). Green lines indicate mean values. RNA transcripts per million (TPM) data for mCRPC patients (total 210 samples from the West Coast Dream Team) were downloaded from https://quigleylab.ucsf.edu/data. **e** Representative images of IHC staining using indicated antibodies on adjacent tissues of $hHGFtg{:}H11^{hMET/+}{:}PB^{Cre4}$ prostates. **f** Representative images of co-IF staining for β-catenin (β-cat) or XPO1 with SP1 on prostate tissues from $hHGFtg{:}H11^{hMET/+}{:}PB^{Cre4}$ mice. Nuclei were stained with DAPI. **g** Representative images of IHC staining using indicated antibodies on adjacent sections of organoids, in presence (top) or absence (bottom) of DHT, derived from $hHGFtg{:}H11^{hMET/+}{:}Ctnnb1^{L(Ex3)/+}{:}PB^{Cre4}$ prostates. See Methods section. Representative images from three biologically independent experiments are shown. Scale bars, 25 μm (**a**, **e**, and **f**-**g**). In **b**-**d**, *$P < 0.05$, **$P < 0.01$, ***$P < 0.001$; Wilcoxon Rank Sum tests.

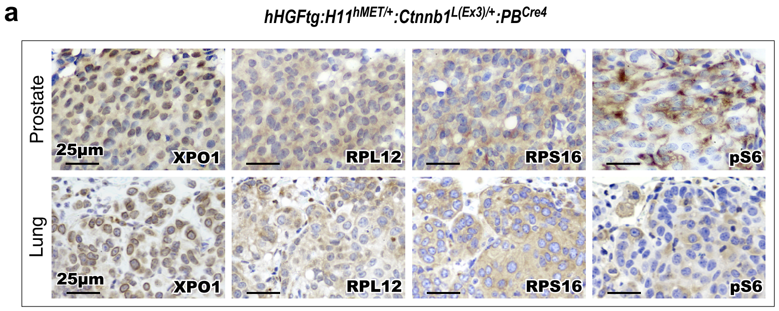

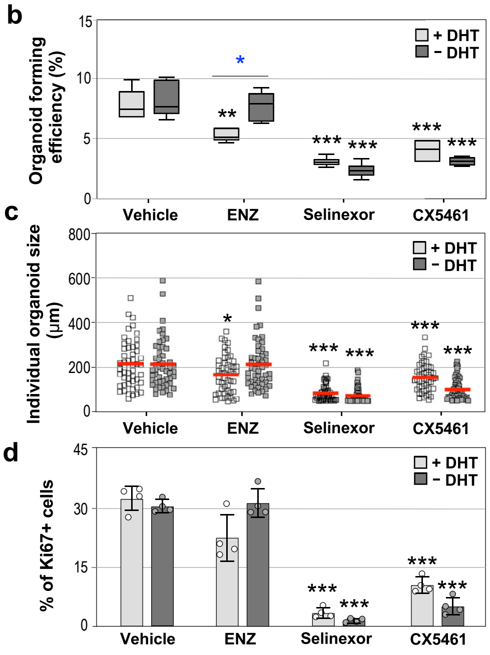

Supplementary Fig. 6 (Related to Fig. 6) Activated nuclear export signal and ribosomal biogenesis induces androgen-independent prostate cancer growth and metastasis. a Representative images of IHC staining using indicated antibodies on adjacent tissues of prostates (top) and lung metastases (bottom) from *$hHGFtg:H11^{hMET/+}:Ctnnb1^{L(Ex3)/+}:PB^{Cre4}$* mice. Representative images with consistent results from at least three biologically independent experiments are shown. Scale bars, 25 μm. **b**-**d** Organoids derived from prostate cancer cells of castrated *$hHGFtg:H11^{hMET/+}:Ctnnb1^{L(Ex3)/+}:PB^{Cre4}$* mice were treated with vehicle, 10 μM Enzalutamide (ENZ), 0.5 μM Selinexor, or 1 μM CX5461 in presence or absence of dihydrotestosterone (DHT) for 6 days. See Methods section. Quantification of organoid forming efficiency showing the percentage of organoids above 50 μm diameter per total cells seeded at day 0 in a well. The center line represents the median value; the box borders represent the lower and upper quartiles (25% and 75% percentiles, respectively); the ends of the bottom and top whiskers represent the minimum and maximum values, respectively (**b**). Quantification of individual organoid size. Organoids per treatment group (n = 50) examined over three biologically independent experiments. The center red bar indicates the mean value in each group (**c**). Quantification of Ki67+ cells per total cells from groups treated as indicated (**d**). Data are mean ± s.d. of four biological replicates. Black stars showed a significant difference between vehicle- and drug-treated samples, and blue stars showed a significant difference between samples in the presence and absence of DHT, *$P < 0.05$, **$P < 0.01$, ***$P < 0.001$; Unpaired two-tailed *t*-tests. Source data and the exact *P* value are provided in the Source Data file.

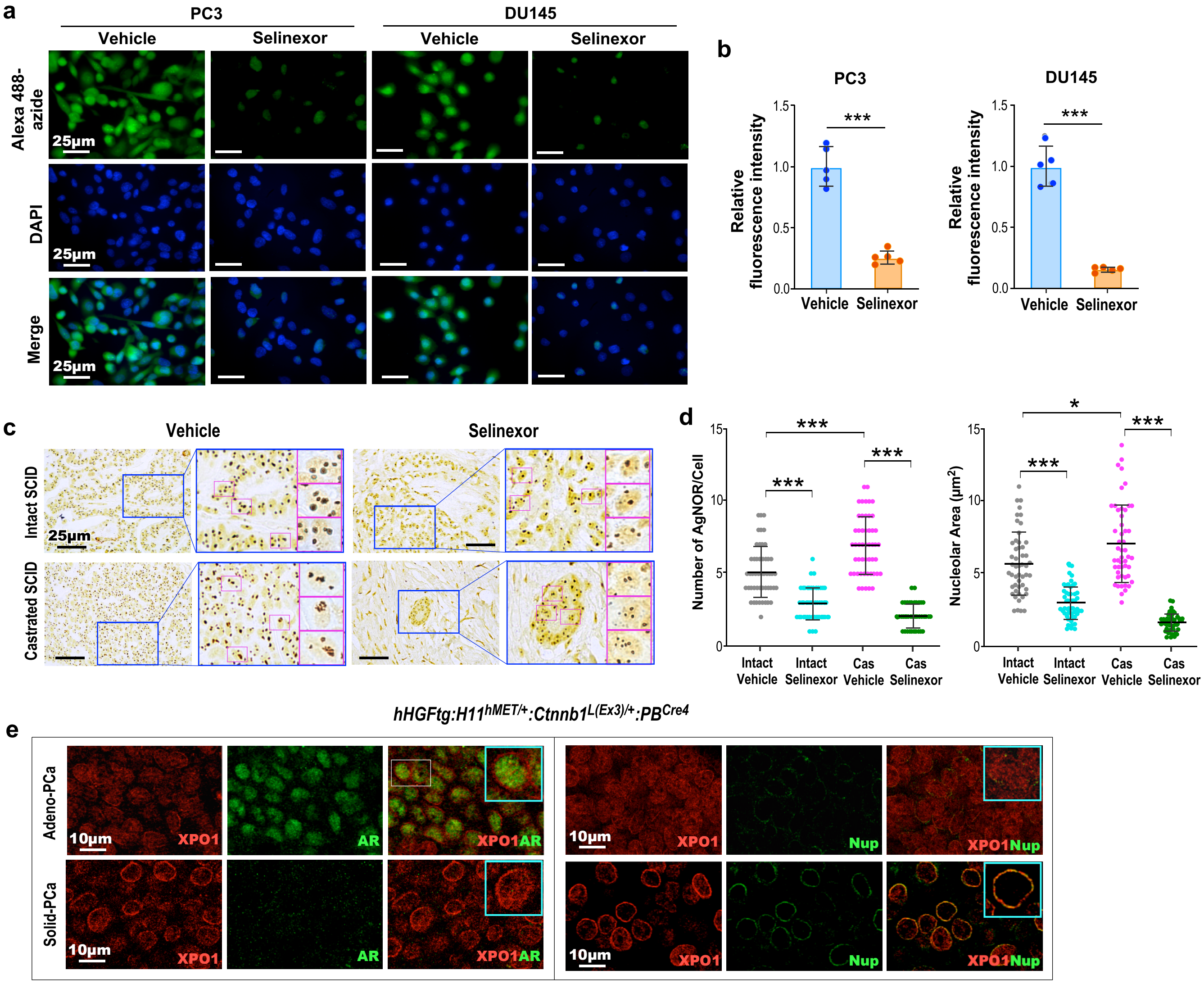

Supplementary Fig. 7 (Related to Fig. 6) Inhibition of XPO1 reduces protein synthesis and nucleolar size in human and mouse prostate cancer cells. a Fluorescence microscopy imaging of global protein synthesis in PC3 and DU145 cells treated with vehicle or 1 μM Selinexor for 24 h. Fluorescence staining of nascent polypeptides was done with O-propargul-puromycin (OPP) using Alexa 488-azide (green) along with DAPI (blue). **b** Quantification of relative Alexa 488 fluorescence intensity based on five areas containing 1000 cells per group from three independent experiments. **c** Representative images of AgNOR stained xenografts from indicated group. Intact and castrated SCID host mice transplanted with dissected prostate cells of *hHGFtg:H11*$^{hMET/+}$*:Ctnnb1*$^{L(Ex3)/+}$*:PB*^{Cre4} mice were administrated with vehicle or Selinexor. See Methods section. **d** Quantification of AgNOR number (left) and nucleolar area (right) in individual cells from the indicated loci. Numbers of AgNORs per cells and nucleolar area were measured for 50 cells in each image, and at least 5 images from three biological replicates were analyzed. **e** Representative images of co-IF staining using indicated antibodies on adjacent prostate tissues from *hHGFtg:H11*$^{hMET/+}$*:Ctnnb1*$^{L(Ex3)/+}$*:PB*^{Cre4} mice. Nups (NUP62, NUP98, and NUP214) were detected by mAb414 antibody in the indicated loci from prostate tissues. In **a**, **c**, and **e**, representative images from three biologically independent experiments are shown. In **b** and **d**, data are mean ± s.d. of three biological replicates. *P < 0.05, ***P < 0.001; Unpaired two-tailed *t*-tests. Scale bars, 25 µm (**a**, **c**), 10 µm (**e**). Source data and the exact *P* values are provided in the Source Data file.

Supplementary Table 1. Information of oligonucleotide primers used in this study.

	Gene	Primer	Sequences
Genotyping	*hHGFtg*	Forward	5' - AGT CTG TGA CAT TCC TCA GTG - 3'
		Reverse	5' - TGA GAA TCC CAA CGC TGA CA - 3'
	H11	Forward	5' - CCT TCA GCT GCC CAC TCT AC - 3'
		Reverse	5' - ACA TCG GTA ATC CAG TGC CC - 3'
	hMETtg	Forward	5' - TGA CCA GTG GGA CTG CTT TTT - 3'
		Reverse	5' - CAC ACG ACC AGG CCT TCC TTC TT - 3'
	Ctnnb1$^{L(Ex3)}$	Forward	5' - AAC TGG CTT TTG GTG TCG GG - 3'
		Reverse	5' - TCG GTG GCT TGC TGA TTA TTT C - 3'
	Pb^{Cre4}	Forward	5' - GAT CCT GGC AAT TTC GGC TAT - 3'
		Reverse	5' - GCA GGA AGC TAC TCT GCA CCT TG - 3'
qPCR	*Ppia*	Forward	5' - TGT GCC AGG GTG GTG ACT TT - 3'
	(mouse)	Reverse	5' - CGT TTG TGT TTG GTC CAG CAT - 3'
	Cd44	Forward	5' - TCG ATT TGA ATG TAA CCT GCC G - 3'
	(mouse)	Reverse	5' - CAG TCC GGG AGA TAC TGT AGC - 3'
	Sox9	Forward	5' - GCG TCA ACG GCT CCA GCA AGA - 3'
	(mouse)	Reverse	5' - GCC AGC TTG CAC GTC GGT TTT G - 3'
	Mmp7	Forward	5' - ACT TCA GAC TTA CCT CGG ATC G - 3'
	(mouse)	Reverse	5' - TCC CCC AAC TAA CCC TCT TGA - 3'
	Plaur	Forward	5' - GTG CCC TCG TGT TGT CTT CT - 3'
	(mouse)	Reverse	5' - CAG CCC TTG TTC CAA TTC TC - 3'
	Myc	Forward	5' - CCC TAT TTC ATC TGC GAC GAG - 3'
	(mouse)	Reverse	5' - TGG GAA GCA GCT CGA ATT T- 3'
	Xpo1	Forward	5' - AGT GCC TCA CTG AGA TTG CTG G - 3'
	(mouse)	Reverse	5' - CCC ATT CGA GTA TGC AAG TCG G - 3'
	Rpl12	Forward	5' - ACT GGA AGG GTC TCA GAA TTA CA - 3'
	(mouse)	Reverse	5' - TGC CGG GCA ATG TTG ACA A - 3'
	Rps16	Forward	5' - GCT CAT CAA GGT GAA CGG ACG T - 3'
	(mouse)	Reverse	5' - ATC CAC ACC AGC AAA TCG CTC C - 3'
	Eif4a1	Forward	5' - ATG TCT GCG AGT CAG GAT TCT - 3'
	(mouse)	Reverse	5' - AGC TAT CCA CAA TCT CGT TCC A - 3'
	18S rRNA	Forward	5' - GGG AGC CTG AGA AAC GGC - 3'
	(human)	Reverse	5' - GGG TCG GGA GTG GGT AAT TT - 3'
	SP1	Forward	5' - ACG CTT CAC ACG TTC GGA TGA G - 3'
	(human)	Reverse	5' - TGA CAG GTG GTC ACT CCT CAT G- 3'
	XPO1	Forward	5' - CTA CAT CTG CCT CTC CGT TGC T- 3'
	(human)	Reverse	5' – CCA ATA CTT CCT CTG GTT TAG CC- 3'
ChIP-qPCR	*Untr4*	Forward	5' - CTC CCT CCT GTG CTT CTC AG - 3'
		Reverse	5' - AAT GAA CGT GTC TCC CAG AA - 3'
	Xpo1	Forward	5' - GGC GCT CCG ACC AAT AAG AG - 3'
	binding site A	Reverse	5' - TCG GTT GAG TTT TCA GCC CAT - 3'
	Xpo1	Forward	5' - GGT TCC GAG TTT GAG GCA CTA - 3'
	binding site B	Reverse	5' - GTT GCT CTT GCT GAT GCT GA - 3'

Supplementary Table 2. Information for antibodies used in this study.

Antibody	Vendors and Cat #	Species	Working dilution
E-cadherin	BD Transduction Laboratories #C20820	mouse IgG	1:200
Synaptophysin	Invitrogen # MA5-14532	rabbit IgG	1:100
AR	ThermoFisher #PA1-9005	goat IgG	1:500
pMET	Cell Signaling #3077	rabbit IgG	1:200
β-catenin	BD Transduction Laboratories #610154	mouse IgG	1:200
MET	ThermoFisher #37-0100	mouse IgG	1:100
mAB414 (Nups)	Abcam #ab24609	mouse IgG	1:200
XPO1	NovusBio #NB100-79802	rabbit IgG	1:1000(IHC/IF) 1:5000(WB)
MYC	Abcam #ab168727	rabbit IgG	1:100
RPL12	Abcam #ab127533	rabbit IgG	1:200
RPS16	Proteintech #15603-1-AP	rabbit IgG	1:250
pS6	Cell Signaling #2211	rabbit IgG	1:100
SP1	Santa Cruz #sc-59	goat IgG	1:50
SP1	NovusBio #NB600-233	rabbit IgG	1:5000
Ki67	Cell Signaling #9129	rabbit IgG	1:500
pGSK3	Cell Signaling #9331	rabbit IgG	1:200
Vimentin	BioLegend #919101	chicken IgG	1:2000
Actin	Sigma Aldrich # A4700	mouse IgG	1:1000
Biotinylated anti-mouse	Vector Laboratories #BA-9200	goat IgG	1:750
Biotinylated anti-rabbit	Vector Laboratories #BA-1000	goat IgG	1:750
Biotinylated anti-goat	Vector Laboratories #BA-5000	rabbit IgG	1:750
Donkey anti-rabbit 488	Invitrogen #A21206	donkey IgG	1:500
Donkey anti-mouse 488	Invitrogen #A21202	donkey IgG	1:500
Donkey anti-rabbit 594	Invitrogen #A21207	donkey IgG	1:500
Donkey anti-mouse 594	Invitrogen #A21203	donkey IgG	1:500
Donkey anti-goat 647	Invitorgen #A21447	donkey IgG	1:500
Goat anti-mouse HRP	Bio-rad #170-6516	goat IgG	1:1000
Goat anti-rabbit HRP	Bio-rad #170-6515	goat IgG	1:1000
SP1 for ChIP	NovusBio #NB600-233	rabbit IgG	1:100
