# Supplementary material for: Androgen deprivation induces double-null prostate cancer via aberrant nuclear export and ribosomal biogenesis through HGF and Wnt activation
Source: Nat Commun. 2024 Feb 9;15:1231. doi: 10.1038/s41467-024-45489-4 (PMC10858246; doi:10.1038/s41467-024-45489-4)
Supplement: Supplementary file 3 — Description of Additional Supplementary Files [file 41467_2024_45489_MOESM3_ESM.pdf]

Description of Additional Supplementary Files

Title: **Supplementary Data 1**
Description: Related to Fig. 1b and Supplementary Fig. 1b,c; DEGs between DNPC or NEPC versus ARPC

Title: **Supplementary Data 2**
Description: Related to Fig. 2g-h and Supplementary Fig. 2l; DEG List between *hMETtg*+ vs *hMETtg*- in mouse prostate epithelial cells of indicated GEMMs.

Title: **Supplementary Data 3**
Description: Related to Fig. 4c and Supplementary Fig. 4i; DEG List between *hMETtg*+ epithelial cells from *hHGFtg:H11*$^{hMET/+}$*:Ctnnb1*$^{L(Ex3)/+}$*:PB*^{Cre4} versus those of *hHGFtg:H11*$^{hMET/+}$*:PB*^{Cre4} mice

Title: **Supplementary Data 4**
Description: Related to Fig. 4j; DEG List between LE2 vs other LE cell clusters from *hHGFtg:H11*$^{hMET/+}$*:Ctnnb1*$^{L(Ex3)/+}$*:PB*^{Cre4} mice

Title: **Supplementary Data 5**
Description: Related to Fig. 4j; DEG List between LE3 vs other LE cell clusters from *hHGFtg:H11*$^{hMET/+}$*:Ctnnb1*$^{L(Ex3)/+}$*:PB*^{Cre4} mice

Title: **Supplementary Data 6**
Description: Related to Fig. 4j; DEG List between LE4 vs other LE cell clusters from *hHGFtg:H11*$^{hMET/+}$*:Ctnnb1*$^{L(Ex3)/+}$*:PB*^{Cre4} mice

Title: **Supplementary Data 7**
Description: Related to RNAseq data in Fig. 7d-e; DEGs list between castrated and intact prostate samples of *hHGFtg:H11*$^{hMET/+}$*:Ctnnb1*$^{L(Ex3)/+}$*:PB*^{Cre4} mice
