# Supplementary material for: Androgen deprivation induces double-null prostate cancer via aberrant nuclear export and ribosomal biogenesis through HGF and Wnt activation
Source: Nat Commun. 2024 Feb 9;15:1231. doi: 10.1038/s41467-024-45489-4 (PMC10858246; doi:10.1038/s41467-024-45489-4)
Supplement: Supplementary file 11 — Reporting Summary [file 41467_2024_45489_MOESM11_ESM.pdf]

nature portfolio

Corresponding author(s): Zijie Sun MD, PhD

Last updated by author(s): Dec 21, 2023

Reporting Summary

Nature Portfolio wishes to improve the reproducibility of the work that we publish. This form provides structure for consistency and transparency in reporting. For further information on Nature Portfolio policies, see our Editorial Policies and the Editorial Policy Checklist.

Statistics

For all statistical analyses, confirm that the following items are present in the figure legend, table legend, main text, or Methods section.

n/a	Confirmed	
☐	☒	The exact sample size (*n*) for each experimental group/condition, given as a discrete number and unit of measurement
☐	☒	A statement on whether measurements were taken from distinct samples or whether the same sample was measured repeatedly
☐	☒	The statistical test(s) used AND whether they are one- or two-sided<br>*Only common tests should be described solely by name; describe more complex techniques in the Methods section.*
☒	☐	A description of all covariates tested
☐	☒	A description of any assumptions or corrections, such as tests of normality and adjustment for multiple comparisons
☐	☒	A full description of the statistical parameters including central tendency (e.g. means) or other basic estimates (e.g. regression coefficient) AND variation (e.g. standard deviation) or associated estimates of uncertainty (e.g. confidence intervals)
☐	☒	For null hypothesis testing, the test statistic (e.g. *F*, *t*, *r*) with confidence intervals, effect sizes, degrees of freedom and *P* value noted<br>*Give P values as exact values whenever suitable.*
☒	☐	For Bayesian analysis, information on the choice of priors and Markov chain Monte Carlo settings
☒	☐	For hierarchical and complex designs, identification of the appropriate level for tests and full reporting of outcomes
☐	☒	Estimates of effect sizes (e.g. Cohen's *d*, Pearson's *r*), indicating how they were calculated

Our web collection on statistics for biologists contains articles on many of the points above.

Software and code

Policy information about availability of computer code

Data collection	Illumina Novaseq 6000 and 10× Genomics Cell Ranger pipeline (6.0.2) were used for single-cell sequencing data collection. The gene expression in our RNAseq data was determined experimentally using the Illumina HiSeq 2500 RNA Sequencing platform. Electron microscopy (EM) images were collected with an FEI Tecnai 12 transmission electron microscope (Thermo Fisher Scientific) equipped with a LaB6 filament and operated at an acceleration voltage of 120 kV. Images were taken with a Gatan Ultrascan 2K charge-coupled device camera (Gatan). Images of H&E and IHC staining were taken by an Axio Lab 1 microscope using 10×, 20×, and 40× Zeiss A-Plan objectives and were captured using a Canon EOS 1000D camera and AxioVision software (Carl Zeiss). Images of IF staining and organoids were acquired on a Nikon ECLIPSE E800 epi-fluorescence microscope at 10×, 20×, and 40× Nikon Plan Fluor objectives using an QImaging RETGA EXi camera with QCapture software (QImaging). Fluorescence images for nuclear pore complex were collected using Zeiss LSM-700 confocal microscope with Zen 2012 Imaging Software.
Data analysis	For single-cell sequencing data analysis, the Seurat package (4.3.0) in the software R (4.2.2) was used. For RNA sequencing data analysis, the filtered reads were aligned to the mm10 mouse reference genome using STAR (v2.7.9), and read counts per gene were detected using HTSeq (v2.0.2) and were further normalized using the trimmed mean of M-value method in edgeR (v2.26.7) to obtain counts per million-mapped reads (CPM) and transcripts per million (TPM). Pathway analysis was performed using Gene Set Enrichment Analysis (GSEA 4.3.2; Subramanian A, et al. Proc Natl Acad Sci U S A 102, 15545-15550 (2005)). GraphPad Prism software v6 was used to generate graphs. ImageJ was used to analyze counting cells, number and size of nucleolus and organoid culture experiment images. R scripts used to process sequencing data are available at "DOI: 10.5281/zenodo.10419988". Included in "Methods" section.

For manuscripts utilizing custom algorithms or software that are central to the research but not yet described in published literature, software must be made available to editors and reviewers. We strongly encourage code deposition in a community repository (e.g. GitHub). See the Nature Portfolio guidelines for submitting code & software for further information.

Data

Policy information about availability of data

All manuscripts must include a data availability statement. This statement should provide the following information, where applicable:

- Accession codes, unique identifiers, or web links for publicly available datasets
- A description of any restrictions on data availability
- For clinical datasets or third party data, please ensure that the statement adheres to our policy

RNA-seq processed data for metastatic prostate adenocarcinoma patients (SU2C/PCF Dream Team, 266 samples; polyA assay samples) and drug treatment status per sample were downloaded from the cBioPortal website (http://www.cbioportal.org/). RNA transcripts per million (TPM) data for mCRPC patients (total 210 samples from the West Coast Dream Team) were downloaded from https://quigleylab.ucsf.edu/data. scRNA-seq datasets from both human naïve primary PCa and ADT-treated mCRPC tissues, which have been deposited in NCBI's SRA database and is accessible through SRA accession: PRJNA699369.
Raw data of RNA-seq and single cell RNA-seq have been publishly deposited in Gene Expression Omnibus database under accession number GSE226556 [https://www.ncbi.nlm.nih.gov/geo/query/acc.cgi?acc=GSE226556]. Source Data for this study are provided with this paper. All relevant data in this study are available within the article, Supplementary information, or Source Data. Statement was included in "Methods" section.

Research involving human participants, their data, or biological material

Policy information about studies with human participants or human data. See also policy information about sex, gender (identity/presentation), and sexual orientation and race, ethnicity and racism.

Reporting on sex and gender	All of the prostate cancer patients were of male sex, the gender identity of each of the participants was not recored in this study.
Reporting on race, ethnicity, or other socially relevant groupings	Socially constructed or socially relevant categorization variables were not used in this study.
Population characteristics	Human prostate cancers included five specimens of prostatectomies from 5 patients without hormonal treatment, and the metastatic CRPC specimens from 6 individual patients who had previously received second line antiandrogen therapies- ENZ and ABI- for at least 3 months after the failure of first line ADT treatment, including two samples from lymph node, three from brain, and one from adrenal gland. They were of high tumor purity. Human prostate cancer tissue samples were collected previously without convariate-relevant characteristics including identifiable personal and private information.
Recruitment	Informed consent was obtained from all patients. Also, patients were asked if they would be willing to donate samples. Biases may include the willingness of patients to donate samples, which likely resulted in minimal impact on this study.
Ethics oversight	The study was approved by the Institutional Review Board (IRB)-approved protocol (IRB # HS-16-00817) at the University of Southern California.

Note that full information on the approval of the study protocol must also be provided in the manuscript.

Field-specific reporting

Please select the one below that is the best fit for your research. If you are not sure, read the appropriate sections before making your selection.

[x] Life sciences   [ ] Behavioural & social sciences   [ ] Ecological, evolutionary & environmental sciences

For a reference copy of the document with all sections, see nature.com/documents/nr-reporting-summary-flat.pdf

Life sciences study design

All studies must disclose on these points even when the disclosure is negative.

Sample size	Sample size estimated in this study was based on number of available, statistical methods, and previous reported data with related mouse models. Sample sizes were also based on reproducibility of the results and in which statistical significance using unpaired two-sided Student's t-test was observed and are consistent with our previous works and publications in the field (Le V, et al. Loss of androgen signaling in mesenchymal sonic hedgehog responsive cells diminishes prostate development, growth, and regeneration. PLoS Genet 16, e1008588 (2020), Kim WK, et al. Aberrant androgen action in prostatic progenitor cells induces oncogenesis and tumor development through IGF1 and Wnt axes. Nat Commun 13, 4364 (2022)). At least three mice per condition were used. This represents a >98% probability of detecting a significant change if alpha is set at 0.05 and SDs are 8% of average. All representative images with consistent results from at least three biological replicates are shown to show statistical significance.
Data exclusions	None of the were data excluded. All of generated male mice were included in the study due to the nature of the prostate cancer models.
Replication	All experiments were performed at least three independent times and/or with sufficient samples per group for statistical significance. All replication attempts were successful.
Randomization	All of experimental mice with different genotypes were included without selection in this study. Negative control groups such as genotype or vehicle treatment were included in all experiments. For in vitro and ex vivo experiments, cells were randomly allocated into control and

experimental groups. For other experiments and analysis, randomization and group allocation is not relevant or applicable in this study as all experimental mice were included based on each genotype from age-matched littermates and were all under the same conditions in each comparison.

Blinding

Blinding methods were used for assessing the pathological changes in prostate tumor developments of each genotype/mouse model used in this study, performing staining experiments, and quantifying all images. For other group allocation, data collection, and analysis, investigators were not blinded to ensure genotype, treatment, and comparison which is not readily subject to investigator bias. Also, all data were collected and analyzed by multiple investigators.

Reporting for specific materials, systems and methods

We require information from authors about some types of materials, experimental systems and methods used in many studies. Here, indicate whether each material, system or method listed is relevant to your study. If you are not sure if a list item applies to your research, read the appropriate section before selecting a response.

Materials & experimental systems

n/a	Involved in the study
☐	☒ Antibodies
☐	☒ Eukaryotic cell lines
☒	☐ Palaeontology and archaeology
☐	☒ Animals and other organisms
☒	☐ Clinical data
☒	☐ Dual use research of concern
☒	☐ Plants

Methods

n/a	Involved in the study
☒	☐ ChIP-seq
☒	☐ Flow cytometry
☒	☐ MRI-based neuroimaging

Antibodies

Antibodies used

Included in Extended Data Table 2 and referred in methods section. All antibodies for immunohistochemistry (IHC), immunofluorescence (IF), and chromatin immunoprecipitation-qPCR used were from commercial sources:
E-cadherin BD Transduction Laboratories #C20820 mouse IgG 1:200,
Synaptophysin Invitrogen # MA5-14532 rabbit IgG 1:100,
AR ThermoFisher #PA1-9005 goat IgG 1:500,
pMET Cell Signaling #3077 rabbit IgG 1:200,
β-catenin BD Transduction Laboratories #610154 mouse IgG 1:200,
MET ThermoFisher #37-0100 mouse IgG 1:100,
mAB414 (Nups) Abcam #ab24609 mouse IgG 1:200,
XPO1 NovusBio #NB100-79802 rabbit IgG 1:1000 (IHC/IF), 1:5000 (WB),
MYC Abcam #ab168727 rabbit IgG 1:100,
RPL12 Abcam #ab127533 rabbit IgG 1:200,
RPS16 Proteintech #15603-1-AP rabbit IgG 1:250,
pS6 Cell Signaling #2211 rabbit IgG 1:100,
SP1 Santa Cruz #sc-59 goat IgG 1:50,
SP1 NobusBio #NB600-233 rabbit IgG 1:5000,
Ki67 Cell Signaling #9129 rabbit IgG 1:500,
pGSK3 Cell Signaling #9331 rabbit IgG 1:200,
Vimentin BioLegend #919101 chicken IgG 1:2000,
Actin Sigma Aldrich #A4700 mouse IgG 1:1000,
Biotinylated anti-mouse Vector Laboratories #BA-9200 goat IgG 1:750,
Biotinylated anti-rabbit Vector Laboratories #BA-1000 goat IgG 1:750,
Biotinylated anti-goat Vector Laboratories #BA-5000 rabbit IgG 1:750,
Donkey anti-rabbit 488 Invitrogen #A21206 donkey IgG 1:500,
Donkey anti-mouse 488 Invitrogen #A21202 donkey IgG 1:500,
Donkey anti-rabbit 594 Invitrogen #A21207 donkey IgG 1:500,
Donkey anti-mouse 594 Invitrogen #A21203 donkey IgG 1:500,
Donkey anti-goat 647 Invitorgen #A21447 donkey IgG 1:500,
Goat anti-mouse HRP Bio-rad #170-6516 goat IgG 1:1000,
Goat anti-rabbit HRP Bio-rad #1706515 goat IgG 1:1000,
SP1 for ChIP NovusBio #NB600-233 rabbit IgG 1:100.

Validation

All antibodies used were from commercial sources and vendor confirmed species reactivity and references listed on vendor website.
E-cadherin https://www.bdbiosciences.com/en-us/products/reagents/microscopy-imaging-reagents/immunofluorescence-reagents/purified-mouse-anti-e-cadherin.610182,
Synaptophysin https://www.thermofisher.com/antibody/product/Synaptophysin-Antibody-clone-SP11-Monoclonal/MA5-14532?imageId=83914,
AR https://www.thermofisher.com/antibody/product/Androgen-Receptor-Antibody-Polyclonal/PA1-9005,

pMET https://www.cellsignal.com/products/primary-antibodies/phospho-met-tyr1234-1235-d26-xp-rabbit-mab/3077,
β-catenin https://www.bdbiosciences.com/en-us/products/reagents/microscopy-imaging-reagents/immunofluorescence-reagents/purified-mouse-anti-catenin.610154,
MET https://www.thermofisher.com/antibody/product/c-Met-Antibody-clone-3D4-Monoclonal/37-0100,
mAB414 (Nups) https://www.abcam.com/products/primary-antibodies/nuclear-pore-complex-proteins-antibody-mab414-ab24609.html,
XPO1 https://www.novusbio.com/products/crm1-antibody_nb100-79802,
MYC https://www.abcam.com/products/primary-antibodies/c-myc-antibody-y69-chip-grade-bsa-and-azide-free-ab168727.html,
RPL12 https://www.abcam.com/products/primary-antibodies/rpl12-antibody-ab127533.html,
RPS16 https://www.ptglab.com/products/RPS16-Antibody-15603-1-AP.htm,
pS6 https://www.cellsignal.com/products/primary-antibodies/phospho-s6-ribosomal-protein-ser235-236-antibody/2211?N=4294960384&fromPage=plp&gclid=Cj0KCQiAjbagBhD3ARIsANRrqEsHyUEVqsyKYf2itS4o1d-u8JLUlDLb_2smDw7j15e8PQL4QQVHZT4aAmFMEALw_wcB&gclsrc=aw.ds,
SP1 https://www.scbt.com/p/sp1-antibody-pep-2,
SP1 https://www.novusbio.com/products/sp1-antibody_nb600-233,
Ki67 https://www.cellsignal.com/products/primary-antibodies/ki-67-d3b5-rabbit-mab/9129,
pGSK3 https://www.cellsignal.com/products/primary-antibodies/phospho-gsk-3a-b-ser21-9-antibody/9331,
Vimentin https://www.biolegend.com/en-us/products/purified-anti-vimentin-antibody-11598?GroupID=GROUP26,
Actin https://www.sigmaaldrich.com/US/en/product/sigma/a4700,
Biotinylated anti-mouse Vector Laboratories https://vectorlabs.com/biotinylated-goat-anti-mouse-igg-antibody.html,
Biotinylated anti-rabbit Vector Laboratories https://vectorlabs.com/biotinylated-goat-anti-rabbit-igg-antibody.html,
Biotinylated anti-goat Vector Laboratories https://vectorlabs.com/biotinylated-rabbit-anti-goat-igg-antibody.html,
Donkey anti-rabbit 488 https://www.thermofisher.com/antibody/product/Donkey-anti-Rabbit-IgG-H-L-Highly-Cross-Adsorbed-Secondary-Antibody-Polyclonal/A-21206,
Donkey anti-mouse 488 https://www.thermofisher.com/antibody/product/Donkey-anti-Mouse-IgG-H-L-Highly-Cross-Adsorbed-Secondary-Antibody-Polyclonal/A-21202,
Donkey anti-rabbit 594 https://www.thermofisher.com/antibody/product/Donkey-anti-Rabbit-IgG-H-L-Highly-Cross-Adsorbed-Secondary-Antibody-Polyclonal/A-21207,
Donkey anti-mouse 594 https://www.thermofisher.com/antibody/product/Donkey-anti-Mouse-IgG-H-L-Highly-Cross-Adsorbed-Secondary-Antibody-Polyclonal/A-21203,
Donkey anti-goat 647 https://www.thermofisher.com/antibody/product/Donkey-anti-Goat-IgG-H-L-Cross-Adsorbed-Secondary-Antibody-Polyclonal/A-21447,
Goat anti-mouse HRP https://www.bio-rad.com/en-us/sku/1706516-goat-anti-mouse-igg-h-l-hrp-conjugate?ID=1706516,
Goat anit-rabbit HRP https://www.bio-rad.com/en-us/sku/1706515-goat-anti-rabbit-igg-h-l-hrp-conjugate?ID=1706515,
SP1 for ChIP https://www.novusbio.com/products/sp1-antibody_nb600-233.
Antibodies were further validated by our own IHC, IF, Western blotting and ChIP prior to experiments.

Eukaryotic cell lines

Policy information about cell lines and Sex and Gender in Research

Cell line source(s)	PC3 and DU145, human male prostate cancer cell lines from the American Tissue Culture Collection (ATCC, CRL-1435 and HTB-81).
Authentication	PC3 https://www.atcc.org/products/crl-1435, DU145 https://www.atcc.org/products/htb-81. Both PC3 and DU145 were authenticated by the cell bank using DNA profile (STR) and cytogenetic analysis.
Mycoplasma contamination	We confirmed all cell lines used in this study tested negative for mycoplasma contamination.
Commonly misidentified lines (See ICLAC register)	No commonly misidentified cell lines were used in this study.

Animals and other research organisms

Policy information about studies involving animals; ARRIVE guidelines recommended for reporting animal research, and Sex and Gender in Research

Laboratory animals

All information for laboratory animals used are included in the methods section. Mice housing conditions are under a 12 h light/dark cycle at 20-24 °C and 30-70% humidity at City of Hope Parvin Animal Facility. They are housed in ventilated cage racks with free access to food and water. Experimental mice generated in this study were mixed from C57BL/6 and C3H SCID backgrounds. The human HGF transgenic (hHGFtg) mice 36 were purchased from the Jackson Laboratory (strain #: 030205). To generate the human MET transgenic (H11hMET) mouse line, we used integrase-mediated transgenesis technology as shown before 37. In brief, human MET cDNA was sub-cloned into the pB378 vector containing an attB recombination site. A loxP-PGK-Neomycin- STOP-loxP cassette was inserted between the CAG promoter and a flag-tagged human MET coding sequence followed by a polyadenylation signal. The DNA was purified and micro-injected along with ϕC31 integrase mRNA into zygotic pro-nuclei of an FVB mouse that contains attP docking site at H11 locus. Correctly targeted mice were screened by mouse tail tissue genomic PCR using P1 (5’-TGACCAGTGGGACTGCTTTTT-3’) and P3 (5’-CACACGACCAGGCCTTCCTTCTT-3’) primers, and further confirmed by DNA sequencing. PB-Cre4 (PBCre4) mice were obtained from the NCI mouse repository (strain #: 01XF5). Mice containing the conditional Ctnnb1 allele (Ctnnb1L(Ex3)) were kindly gifted from Dr. Makoto M. Taketo. Human HGFtg:H11hMET/+ female mice were first generated by intercrossing hHGFtg males with H11hMet/hMet female mice and then mated with PBCre4 males to generate H11hMET/+:PBCre4 and hHGFtg:H11hMET/+:PBCre4 littermates at age of 3, 6, and 10 months. Ctnnb1L(Ex3)/+:PBCre4 were generated by intercrossing

Ctnnb1L(Ex3)/L(Ex3) females with PBCre4 male mice. Similar mating procedures were used to generate hHGFtg:H11hMET/+:Ctnnb1L (Ex3)/+:PBCre4 mice at age of 3, 6, and 10 months. Experimental mice generated in this study were mixed from C57BL/6 and C3H SCID backgrounds. 8-12 week-old mice were used for mating in this study. 8-week-old male NOD.CB17-Prkdc(SCID)/_(Jax_001303) mice were used for tissue recombination grafting experiments.

Wild animals

This study did not involve wild animals.

Reporting on sex

We used female mice as breeders and male mice as breeders and experiments.

Field-collected samples

This study did not involve samples collected from the field.

Ethics oversight

All experimental procedures and care of animals in this study were carried out according to the Institutional Animal Care and Use Committee (IACUC) at Beckman Research Institute at City of Hope (California, US), and approved by the IACUC. Euthanasia was performed by CO_2 inhalation followed by cervical dislocation.

Note that full information on the approval of the study protocol must also be provided in the manuscript.

Plants

Seed stocks

Report on the source of all seed stocks or other plant material used. If applicable, state the seed stock centre and catalogue number. If plant specimens were collected from the field, describe the collection location, date and sampling procedures.

Novel plant genotypes

Describe the methods by which all novel plant genotypes were produced. This includes those generated by transgenic approaches, gene editing, chemical/radiation-based mutagenesis and hybridization. For transgenic lines, describe the transformation method, the number of independent lines analyzed and the generation upon which experiments were performed. For gene-edited lines, describe the editor used, the endogenous sequence targeted for editing, the targeting guide RNA sequence (if applicable) and how the editor was applied.

Authentication

Describe any authentication procedures for each seed stock used or novel genotype generated. Describe any experiments used to assess the effect of a mutation and, where applicable, how potential secondary effects (e.g. second site T-DNA insertions, mosiacism, off-target gene editing) were examined.
